# Acute Treatment of Traumatic Tattooing With Dermabrasion: A Case Report

**DOI:** 10.1155/crdm/4084268

**Published:** 2025-08-06

**Authors:** Khalifa Al Alawi, Alreem Al Khayarin, Hanaa Al Kalbani, Sultan Al Shaqsi

**Affiliations:** ^1^Department of Plastic & Reconstructive Surgery, Hamad Medical Cooperation, Doha, Qatar; ^2^Department of Plastic & Reconstructive Surgery, Khoula Hospital, Muscat, Oman

**Keywords:** dermabrasion, scar, traumatic tattooing

## Abstract

Traumatic tattoos result from foreign particles embedding into the dermis, often following industrial accidents or explosions. Among available treatments, including laser therapy and surgical excision, dermabrasion remains a cost-effective and widely accessible option. We present the case of a 49-year-old female construction supervisor who sustained extensive facial traumatic tattooing from the explosion of a hydraulic cement mixer. Clinical examination identified deeply embedded cement particles with localized erythema and edema on the left face. Following stabilization, she underwent acute dermabrasion under general anesthesia, employing staged removal of superficial and partial dermal layers to optimize particle clearance while preserving viable tissue. At 3-month follow-up, the patient demonstrated substantial reduction of pigmentation, minimal scarring, and high satisfaction. Dermabrasion, traditionally applied to superficial dermal lesions, proved particularly effective in this acute context by limiting chronic pigmentation, fibrosis, and textural irregularities. Compared to laser therapy, which requires multiple sessions and carries risk of incomplete clearance in particulate-laden wounds, or surgical excision, which may cause contour deformity, dermabrasion offers immediate, broad-surface intervention with relatively low morbidity. This case underscores dermabrasion's value as a first-line modality for acute traumatic tattoos, especially where resources constrain advanced laser platforms. Prompt recognition and intervention are critical to outcome, and while further research should refine timing, technique, and adjunctive care, dermabrasion remains a pragmatic, effective option in managing acute dermal trauma with embedded particulate matter.

## 1. Introduction

Traumatic tattooing occurs when pigmented particles are accidentally embedded into the dermis. It is broadly classified into 2 categories: abrasive and explosive. Abrasive tattooing occurs when shearing forces abrade the superficial skin layers, concurrently driving foreign particles into the dermis. In contrast, explosive tattooing results from the forceful embedding of particles into the dermis, typically caused by gunpowder explosions [1]. Regardless of the underlying mechanism, the resulting trauma produces an irregularly patterned tattoo, most commonly affecting exposed areas of the skin, such as the upper limbs and face. Several methods have been employed for the treatment of these types of tattoos. These include hydrosurgery, wound debridement using brushes, microsurgical excision, fractional CO_2_ laser therapy, and Q-switched laser treatment [1–4].

We present a case of extensive facial traumatic tattooing successfully treated with dermabrasions. This case highlights the unique use of acute dermabrasion for extensive facial traumatic tattooing, offering a cost-effective and accessible alternative to laser or surgery.

## 2. Case History

A 49-year-old female who works as a construction site supervisor presented following the explosion of a hydraulic cement mixer. She denied any loss of consciousness. The trauma team assessed her and ruled out any concomitant injuries. She was subsequently referred to the plastic surgery team for the evaluation and management of a significant facial traumatic tattooing with cement particles. The patient reported a persistent burning sensation on the left side of her face. She denied any ocular symptoms.

The patient was clinically normal and exhibited no signs of acute distress. Focused examination of the left side of her face revealed numerous minute cement fragments deeply embedded in the dermis, along with swelling and erythema. The area is diffuse and extends from the zygoma down to the mandibular angle. The right sided face is spared. The facial nerve was intact, and an ocular assessment showed no evidence of corneal injury. There were no intraoral or intranasal injuries.

### 2.1. Surgical Management

The patient was taken to the operating room for acute dermabrasion of the traumatic tattoo under general anesthesia with endotracheal intubation. She received perioperative antibiotics and tranexamic acid.

**Figure 1:**
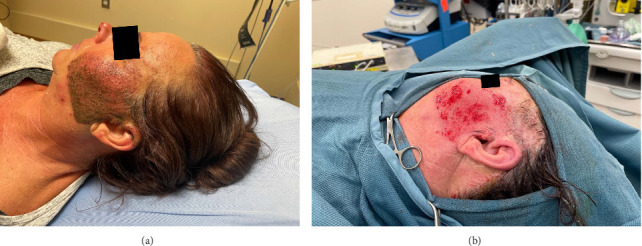
(a) Traumatic tattooing of left side of the face. (b) An intraoperative image of the patient after dermabrasion procedure.

After inducing the patient under general anesthesia, the left side of the face was prepped and draped. The left sided face was initially washed with diluted hydrogen peroxide and saline-soaked gauze, followed by antiseptic painting with Betadine. Gentle saline irrigation was performed over the affected area, and a layer of Vaseline ointment was applied to facilitate dermabrasion. The procedure began using a coarse diamond-tip dermabrader operating at 150, which was applied to the tattooed area in 3-minute passes to remove the superficial epidermal and dermal layers. Punctate bleeding and partial removal of the embedded fragments were observed. This was followed by a fine-tipped dermabrader at 300 RPM for an additional 3 min. Tranexamic acid–soaked gauze was then applied to the treated area to control bleeding. Manual removal of larger embedded cement fragments was performed, and a final refining pass with the dermabrader ensured a smooth dermal contour. Hemostasis was achieved, and the area was covered with a generous application of antibiotic-based ointment and a nonadherent dressing (Figure 1).

### 2.2. Postoperative Course

**Figure 2:**
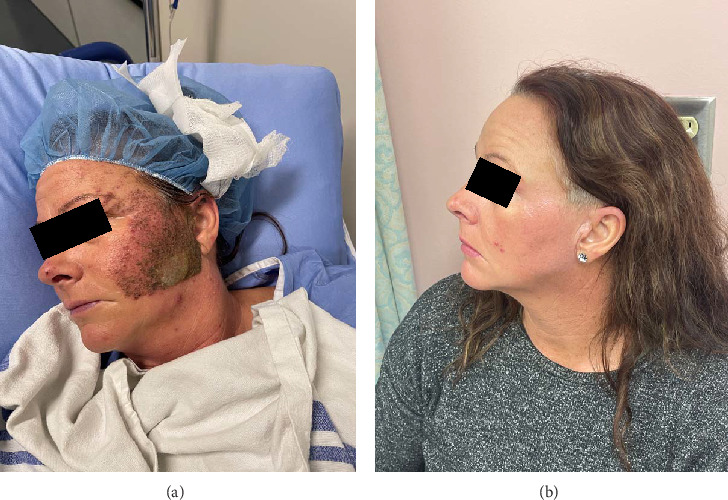
(a) Traumatic tattooing of left side of the face. (b) The results of dermabrasions at 3-month follow-up.

The patient was admitted for overnight observation and discharged on postoperative day 1. Dressings were changed every other day using nonadherent layers. Postprocedural results showed significant improvement, with minimal scarring. By the second week, the patient had fully healed with minimal noticeable scarring. By the third month follow-up, the patient reported high satisfaction with the outcome, particularly the restoration of her skin's appearance, and reported better self-esteem. Regular use of sunscreen and topical moisturizers was recommended to prevent further pigmentation changes and support optimal healing.  Figure 2 compares the preoperative appearance of traumatic tattooing on the left side of the face (a) with the postoperative results following dermabrasion at the 3-month follow-up (b).

## 3. Discussion

Traumatic tattoos occur when foreign particles are embedded in the dermis layer of the skin following a traumatic event. The type of embedded material varies depending on the mechanism of injury, with asphalt being common in motor vehicle accidents and gunpowder or metallic fragments often seen in industrial accidents [5]. In this case, a 49-year-old construction site supervisor presented with traumatic tattooing and superficial burns on the left cheek following the explosion of a hydraulic cement mixer. Embedded asphalt particles raised significant cosmetic and psychological concerns, along with the risk of chronic pigmentation changes and scarring. Since the patient sought medical attention within 48 h of the injury, prompt intervention was essential to prevent deeper embedding of the particles and to mitigate long-term complications such as fibrosis and pigmentation changes.

Dermabrasion is a procedure that involves the use of an abrasive device to remove the superficial, and sometimes deeper, layers of the skin to stimulate regeneration and improve its appearance. The procedure can be performed under local anesthesia with or without sedation, or under general anesthesia [6]. Dermabrasion can be categorized based on the technique into mechanical dermabrasion, microdermabrasion, laser dermabrasion, and cold plasma dermabrasion. Mechanical dermabrasion is skin resurfacing that utilizes a high-speed wire brush, diamond cylinder, fraise, or manual silicon carbide sandpaper. Superficial procedures target the epidermis, while deeper treatments remove both the epidermis and part of the dermis [7, 8]. Given its ability to reach deeper skin layers, mechanical dermabrasion is more suitable for treating deep acne scars, traumatic and surgical scars, actinic keratoses, perioral and periorbital wrinkles, certain congenital lesions (such as epidermal nevi), rhinophyma associated with severe rosacea, and tattoo removal [9]. However, if not performed properly, it can lead to delayed wound healing and even increase the risk of scarring. In addition, mechanical dermabrasion is more suitable for lighter skin types, such as Fitzpatrick types I and II. When used on higher Fitzpatrick skin types, it may increase the risk of postinflammatory hyperpigmentation [10]. On the other hand, microdermabrasion is a noninvasive skin exfoliation procedure that relies on small abrasive particles, such as aluminum oxide crystals, combined with vacuum suction. The device is a closed-loop system where the abrasive material is propelled against the skin surface to create controlled microinjury. At the same time, the vacuum removes spent crystals and exfoliated skin debris, keeping the process clean and minimizing contamination [11, 12]. Due to its limited depth of penetration, microdermabrasion is particularly effective for treating superficial acne scars, mild textural irregularities, photoaging, and enlarged pores. Additionally, it offers a shorter healing time compared to traditional mechanical dermabrasion. However, multiple treatment sessions are often necessary to achieve optimal results [12].

In addition, skin abrasion can be achieved using ablative lasers such as carbon dioxide (CO_2_) or erbium:yttrium-aluminum-garnet (Er:YAG), a technique commonly known as ablative laser resurfacing. The advantage of this approach is its ability to deliver controlled penetration depth, allowing treatment to be precisely tailored to the specific therapeutic target. It is indicated for deep facial wrinkles, more severe acne scars, actinic keratoses, and other forms of photodamage. However, the procedure carries risks including burns, erythema, hyperpigmentation or hypopigmentation, and prolonged healing times [13].

Recently, new technologies have emerged in the field of skin abrasion, such as cold plasma abrasion. In this technique, plasma is generated by ionizing atmospheric air or argon gas, which produces reactive oxygen and nitrogen species. These reactive species are then directed to the skin, creating an ablative effect. The main advantage of cold plasma abrasion is that it causes less thermal injury to the surrounding skin compared to laser ablation. In addition, it has demonstrated antimicrobial effects against a wide range of pathogens, including viruses, bacteria, fungi, and parasites. Although this technology is still under extensive investigation, early studies show promising results in the treatment of superficial acne scars and skin pigmentation disorders [14, 15]. Another form of plasma abrasion is voltaic arc abrasion, which generates plasma by creating a high-frequency electrical arc between the tip of a handheld device and the skin surface [16].

In addition to the abovementioned ablative and abrasive techniques, hydrosurgery systems such as the Versajet offer an alternative approach for the removal of embedded particles. The system operates by delivering a high-pressure jet of saline combined with simultaneous suction, allowing for precise, selective removal of tissue layers and sculpting of soft tissue [17]. Given the depth of the embedded particles in this case and the urgency of treatment, we selected mechanical dermabrasion as an effective method for managing this traumatic tattooing.

Early intervention is essential in acute presentations to prevent particles from embedding deeper into dermal layers. Laser therapy, particularly in chronic tattoos, promises success with minimal scarring but has limitations such as thermal injury, especially in open wounds [4]. Advanced laser techniques, such as fractional resurfacing combined with Q-switched lasers, have demonstrated excellent outcomes with reduced side effects in chronic cutaneous traumatic tattoos [18]. However, the high cost and limited availability of these methods may restrict their widespread application. Similarly, surgical excision, though effective in removing embedded particles, was avoided to minimize scarring and preserve facial aesthetics. Given the acute nature of the presentation and the superficial embedding of particles, dermabrasion was chosen as the treatment modality [7].

A published case report described a facial traumatic tattoo caused by a domestic gas explosion, consisting of superficially embedded, fine carbon particle deposition. Treatment was initiated 6 months after the accident using a Q-switched laser system over 4 sessions, achieving excellent results [19]. In contrast, the use of a laser in our case might not have been ideal due to the larger size of the particles and their deeper embedding. Moreover, it was more convenient for the patient to undergo treatment in a single session rather than multiple sessions.

As this case is descriptive, its findings are limited in their generalizability. Future qualitative studies with larger sample sizes could help guide the acute management of traumatic tattoos and refine treatment protocols. Furthermore, the operator dependence of dermabrasion underscores the importance of skilled execution to prevent overtreatment and potential scarring. Future studies should compare dermabrasion to alternative treatments in acute settings and explore combinations with topical agents to enhance healing. Integrating dermabrasion with advanced technologies and adjunctive care may further improve outcomes. There is a notable lack of literature addressing its use in the acute treatment of traumatic tattooing. Current studies primarily focus on chronic presentations or compare dermabrasion with other modalities for established pigmentation or scarring. This highlights the need for future research to develop standardized acute management protocols.

## 4. Conclusion

In conclusion, this case highlights dermabrasion as a reliable first-line treatment for acute traumatic tattoos, particularly in resource-limited settings. Its accessibility, cost-effectiveness, and ability to achieve significant cosmetic and psychological benefits underscore its continued relevance in clinical practice.

## Data Availability

All the original data generated for this manuscript are available from the corresponding author upon reasonable request.
